# Experimental Study and Numerical Modeling of Thermoviscoelastic Behavior of Antifriction Polymeric Materials

**DOI:** 10.3390/polym18121480

**Published:** 2026-06-12

**Authors:** Anna A. Kamenskikh, Anastasia P. Bogdanova, Yuriy O. Nosov, Yulia S. Kuznetsova

**Affiliations:** 1 Department of Computational Mathematics, Mechanics and Biomechanics, Perm National Research Polytechnic University, 614990 Perm, Russia; anstasia_pankova@mail.ru (A.P.B.); ura.4132@yandex.ru (Y.O.N.); suhodolchik@mail.ru (Y.S.K.); 2 Laboratory of Digital Engineering of Mechanical Engineering Processes and Production, Perm National Research Polytechnic University, 614990 Perm, Russia

**Keywords:** PTFE, UHMWPE, bridge bearing, sliding layer, dynamic mechanical analysis, Prony series, WLF model, finite element analysis, thermoviscoelasticity, contact mechanics

## Abstract

Five modifications of polytetrafluoroethylene (PTFE) are considered as a modern alternative to PTFE as sliding layers of bridge bearing parts. Radiation-modified PTFE without additives and with nano-additives as well as composites based on PTFE with bronze inclusions and nanomodified carbon fiber fillers were investigated. Ultra-high-molecular-weight polyethylene (UHMWPE) and classic pure PTFE were considered as control samples. The thermomechanical properties of the materials were studied within the framework of dynamic mechanical analysis in the operating temperature range of bridge structures [−40; +80] °C. The exit zones from the linear theory of viscoelasticity were established for all the materials considered. Temperature dependencies of the storage modulus and the loss modulus were determined. Thermoviscoelastic models of material behavior were constructed using a numerical identification procedure, experimental data, and simulation models. The thermomechanics of materials during the deformation of the spherical support part of the bridge were analyzed. Temperature dependencies of the parameters of the contact stress-strain state were determined with an average coefficient of determination R^2^ = 0.97 and an average error size RMSE = 0.092.

## 1. Introduction

Polymers are used in various city-forming industries, such as construction [1], transport [2], medicine [3,4], oil and gas industry [5], etc. Changing production standards and solving environmental problems associated with their use led to the development of different types of polymer materials and the expansion of their applications in various industries [6,7,8]. Modern polymer materials differ in properties in different aspects: mechanical, physical, chemical, and technological.

The mechanical properties of polymers depend on the degree of change in their structure, composition, size, and shape, as well as on temperature and mechanical loading [9,10]. The type of filler and its mass content have a significant impact on the thermomechanical behavior of materials [10]. This leads to the need to select materials for the required operating conditions, taking into account temperature factors. It is necessary to evaluate the functional properties of materials when they are used in various designs [11]. Experimental studies are the main ones at the stage of material selection. Heat resistance, mechanical strength, and wear resistance determine the key properties of structural materials. Their applicability in products also depends on the ambient temperature and thermal loads in general [12]. The glass transition temperature is a fundamental characteristic of amorphous and semi-crystalline polymers. It determines the boundaries of the state of the material, from solid to highly elastic. It significantly affects the scope of the material, making it possible to assess the elasticity and flexibility of polymer products according to temperature limits. The transition to the glassy state usually occurs in a wide range of temperatures [13]. The value of the glass transition temperature Tg depends on the heating/cooling rate of the sample, as well as on the complex effect of temperature and load [14,15]. The transition to a highly elastic state changes the processes of friction and wear, as a rule, worsening the strength and wear resistance of the polymer [16,17,18]. Temperature is one of the main factors affecting the properties of polymer and composite materials based on them [19,20]. However, it is the combined effect of temperature and force loads that to a greater extent affects the thermomechanical properties [21].

In this paper, attention is paid to polymer materials used in the bridge construction industry as antifriction layers of thorn nodes (support, bearing). Their work is due to a decrease in friction between steel structural elements, which reduces the wear of parts and increases the service life of bridge structures. The loading rate, strain amplitude, friction coefficient, and temperature regime have been identified as the key parameters affecting the operation of the bearing [22]. The study [23] showed that a decrease in the efficiency of bearings at low temperatures may be the result of the combined effects of low temperature and deformation. Taking into account that the thermal effect and conducting temperature monitoring are extremely important at the design stage of elements of bridge structures [24], in [25], the authors indicate the need to take into account the temperature dependence of the characteristics of the plain bearings under the seismic loads of insulated bridges, since otherwise, the risk of damage will be significantly underestimated. Experimental studies show a significant relationship between the mechanical characteristics of the bearing and the ambient temperature [26], including under cyclic loads [27,28]. In general, temperature has a strong effect on both the properties and the evolution of the stress-strain state of products made of polymer materials [16,29], which in turn determines the main focus of the current study.

Qualitative construction of a numerical analog of the structure, including analysis of the finite element mesh, settings for the convergence of the solution of a nonlinear problem, and the selection of a model of material behavior, is necessary to assess the stress-strain state of important nodes [30]. Conducting a wide range of experiments will make it possible to evaluate the fundamental aspects of the behavior of the material. This will identify areas that affect the performance of the structure from the point of view of their modeling. Accurate knowledge of material properties over a wide temperature range is extremely important in numerical modeling. The accumulation of information on the behavior of materials is necessary to the development of modern methods of assessing and predicting the physical characteristics of polymers [31,32]. Due to the complexity of the mechanisms of deformation of polymer materials, mathematical models of behavior description are also developing quite rapidly. These include elastic-plastic [33], viscoelastic-plastic [34,35], and viscoelastic models [36,37]. The identification of model parameters for numerical implementation is often reduced to an optimization problem [38]. However, there are also modern approaches based on neural networks and machine learning [39,40].

Polytetrafluoroethylene (PTFE) was first synthesized in 1938 by Roy Plunkett, and it is still considered to be the “king of plastics” [41,42]. PTFE (fluoroplastic, Teflon) earned its title of “king” due to its chemical inertness, hydrophobicity, low surface energy, and resistance to thermal, biological, and oxidative decomposition, as well as a low coefficient of friction. Its chemical structure consists of a carbon skeleton surrounded by a protective layer of fluorine atoms [43]. PTFE shows a high degree of structural regularity, which can initiate a greater degree of crystallization of the microstructure, especially at negative temperatures. PTFE exhibits a viscoelastic nature [44]. Plastic deformation of the material, poor thermal stability, and a large coefficient of thermal expansion (CTE) are also noted as features of the thermomechanical behavior of the material [45,46]. The service life of PTFE products, coatings, and interlayers depends on environmental conditions, mechanical stresses, and thermal loads, including cyclic ones, as well as the influence of corrosive media [47].

Progress in the field of materials science has made it possible to form a fairly large set of alternatives to the “king of plastics”. Ultra-high-molecular-weight polyethylenes (UHMWPE) are one of its competitors [48]. Modifications and composites based on PTFE and UHMWPE are also widespread [49,50]. A set of modern antifriction nanomodified and nanofilled materials based on PTFE and produced in Russia and Belarus is considered an alternative to materials in wide use on the sliding layers of bridge bearings.

The proposed method was previously tested on the polymer material UHMWPE [47]. The possibility of using the relations of viscoelasticity theory to describe antifriction polymeric materials over a wide range of operating temperatures was demonstrated. However, at present, there is a need to describe a wide class of antifriction polymeric materials for numerical modeling of the spherical bridge bearing. This paper presents the results of an experimental study to determine the physical and mechanical properties of a set of modern antifriction materials. The parameters of the viscoelastic model with the decomposition of the relaxation core by the Prony series were identified. A numerical analog of the structure of the support part of the bridge under force loading conditions, taking into account the temperature, was built. The results obtained are necessary for future studies of the behavior of the support parts of bridges under cyclic loading. They will make it possible to formulate recommendations for the use of modern polymers as an antifriction layer in critical friction units under various loads and temperatures. This will ensure compliance with the main criteria for the selection of materials at the stage of designing structures.

## 2. Materials and Methods

### 2.1. Materials

Materials of the Arflon line (Scientific and Production Enterprise “Arflon” LLC, Moscow, Russia) are considered an alternative to pure PTFE. They are obtained by compacting pure and/or additive-filled PTFE powder, followed by high-temperature radiation treatment. In this study, three basic brands of Arflon are considered: AR-200 (“Arflon” LLC, Moscow, Russia), AR-202 (“Arflon” LLC, Moscow, Russia), and AR-204 (“Arflon” LLC, Moscow, Russia). All of them are based on PTFE powder and modified according to a single technological mode with a degree (dose) of modification 2. The specific parameters of the technological process are not disclosed by the manufacturer.

AR-200 is a structurally modified PTFE without fillers. Is is a white, translucent material. According to the manufacturer, it has improved wear resistance, radiation resistance, and heat resistance, as well as a decrease in creep and friction coefficient. The material is recommended for tribotechnical products.

Composite materials produced by Arflon contain finely dispersed organic and inorganic fillers. After pressing, they are also subjected to high-temperature physicochemical modification. AR-202 and AR-204 differ in their types of nanofiller—foundry coke and carbon fiber, respectively. The mass fraction of the filler is 20% in both materials, making them black in color. They are also recommended for tribosystem parts.

According to the patent by Artamonov et al. [51], Arflon materials are heated in a thermal chamber above the crystals’ melting point at a pressure of up to 1 mmHg and subjected to gamma irradiation with an average quantum energy of 1.25 MeV to an absorbed dose of no more than 200 kGy, followed by cooling to room temperature. The size of the filler particles is not disclosed.

Composite Superfluvis SF-1, developed by Grodno Mechanical Plant JSC (Grodno, Republic of Belarus) and the Institute of Mechanics of Metal-Polymer Systems (which was named after V. A. Bely of the National Academy of Sciences of Belarus (Gomel, Republic of Belarus)) is considered a promising material for antifriction coatings and layers [50]. The SF-1 material is manufactured on the basis of PTFE using plasma-modified carbon fiber. Approximately 17% of the total weight falls on the modified crushed carbon fiber, the surface of which is coated with a fluoropolymer, forming a nanocoating up to 40 nm thick.

According to the patent by Struk et al. [52], carbon fiber is crushed to size of at least 100 µm, followed by modification with a 1–2% solution of a fluorine-containing oligomer with a molecular weight of 2000–5000 units, drying to remove the solvent, and heat treatment at a temperature of 373 ± 5 K for 1–5 min. The filler is mixed with PTFE powder in specified proportions. More detailed information on the technological process of SF-1 production is not presented in open sources.

Also, for comparative analysis, this article considers three materials widely used as sliding layers of bridge bearing parts: PTFE (AlfaTech LLC Perm, Russia); PTFE-based composite material with 40% bronze dendritic inclusions and 2% molybdenum disulfide (F4Br40M2) (AlfaTech LLC Perm, Russia); and ultra-high molecular weight polyethylene (UHMWPE) (AlfaTech LLC Perm, Russia). Materials were obtained on from the production company AlfaTech LLC (Perm, Russia).

The F4Br40M2 composite contains bronze dendritic inclusions of Rogal Bronze GS 0/40-03 (Carl Schlenk, Germany) with a size of less than 40 μm [49]. The technological process for forming the material involves mixing PTFE powder with filler in a paddle mixer, pressing under loads of up to 40 MPa, and multi-stage sintering at temperatures of up to 370 °C. The technological process is described in detail by Adamov et. al. [49].

All of the materials in question are suitable in one way or another as relatively thin, flat, and spherical sliding layers of bridge bearing parts. The thermomechanical properties of materials require analysis, since the structures operate in a wide range of operating temperatures.

The main properties of the materials, according to manufacturers and/or determined experimentally in previous studies, are summarized in Table 1.

Limited data on the manufacturing processes of antifriction polymer and composite materials is noted. This is largely due to the manufacturers’ trade secrets.

### 2.2. Setting up the Experiment

Dynamic mechanical analysis (DMA) was selected to determine the thermoviscoelastic properties of materials. Rectangular samples L × B × H were studied according to the three-point bending scheme under oscillating loads in the temperature range from −40 °C to +80 °C. DMA Q800 (TA Instruments, New Castle, DE, USA) was used to conduct a series of experiments.

Samples were made by AlfaTech LLC (Perm, Russia) from industrial blanks of sliding layers of bridge bearing parts. The technology for forming industrial blanks used in the production of bridge bearings is disclosed in [49] and includes mixing the material components in a high-speed mixer, pressing at a pressure of 30–50 MPa, sintering in a free state at a temperature of 360–380 °C, followed by cooling in a furnace. Five samples from each material were made for experiments. Geometrical dimensions of the specimens: L = 59.99 ± 0.49 mm, B = 11.95 ± 0.13 mm, and H = 3.08 ± 0.11 mm.

The linear viscoelasticity zone was determined using one sample. This made it possible to establish the amplitudes and deformations near the exit zone from the linear theory of viscoelasticity (Table 2); these amplitudes and deformations were then used in the main experiment.

The experiment was carried out in two stages: (1) heating the sample to +80 °C for 20 min with exposure at a given temperature for 10 min; (2) oscillating load near the exit zone from the linear theory of viscoelasticity with a frequency of 1 Hz with gradual cooling to −40 °C and a rate of change of 2 °C/min.

During the experiment, the temperature dependence of the storage modulus, the loss modulus, and the tangent of the angle of mechanical losses was recorded. For static significance of the results, the experiment was repeated four times for each material. This also provides an analysis of the homogeneity of the properties of the materials in the industrial blanks of the sliding layers.

The choice of constant frequency experimental conditions was driven by the need to obtain a unified experimental dataset for subsequent identification of the Maxwell model parameters and the Williams–Landel–Ferry temperature–time shift function (WLF). This approach allows the DMA results to be used directly in the numerical description of the thermoviscoelastic behavior of materials and to transfer the obtained parameters to the finite element model of the bridge bearing without additional testing.

### 2.3. Constitutive Model of the Material

The general equation of a linear viscoelastic material [53] has the form:

(1) $$\sigma \left(t\right)=\int_{0}^{t}C\left(t-\tau \right):\frac{d\varepsilon \left(\tau \right)}{d\tau }d\tau ,$$

where $\sigma \left(t\right)$ is the stress tensor; $\varepsilon \left(t\right)$ is the strain tensor; and $C\left(t\right)$ is the relaxation tensor, which, unlike the tensor of elastic constants C, describes the time dependence of the material response and takes into account the history of deformation.

As part of the study, it is assumed that the material is isotropic; therefore, $C\left(t\right)$ can be decomposed into shear and bulk parts:

(2) $$C\left(t\right)=2G\left(t\right)I_{d}+K\left(t\right)I⊗I,$$

where $G\left(t\right)$ is the shear modulus; $K\left(t\right)$ is the modulus of volume compression; $I_{d}$ is the tensor of projection on the deviator part of the deformation; I⊗I is the tensor of projection on the volume part of the deformation; and ⊗ is the tensor product.

Any positively defined relaxation function $G\left(t\right)$ and $K\left(t\right)$ can be represented as an integral over the relaxation spectrum:

(3) $$S\left(t\right)=S_{\infty }+\int_{0}^{t}H\left(\tau \right)e^{-t/\tau }d\tau ,$$

where $S_{\infty }$ is the residual (final) modulus and $H\left(\tau \right)$ is the spectral density of relaxation. Such a decomposition $G\left(t\right)$ $K\left(t\right)$ corresponds to a continuous set of Maxwell elements (consecutive connection of the elastic and viscous elements).

Prony series are widely used to approximate the final sum of the integral in modern computer-aided design systems.

(4) $$\int_{0}^{t}H\left(\tau \right)e^{-t/\tau }d\tau \approx \sum_{i=1}^{N}\alpha_{i}S_{0}e^{-t/\beta_{i}},$$

where $\alpha_{i}=\int_{\beta_{i-1}}^{\beta_{i}}H\left(\tau \right)d\tau /S_{0}$ is the relative weight of the *i*-th element; $\beta_{i}$ is the characteristic relaxation time on the *i*-th interval; and N is the number of members of the series.

Based on (3) and (4), the shear modulus (5) and the volumetric compression modulus (6) have the form

(5) $$G\left(t\right)=G_{0}\left[\alpha_{\infty }^{G}+\sum_{i=1}^{N_{G}}\alpha_{i}^{G}e^{-t/\beta_{i}^{G}}\right],$$

(6) $$K\left(t\right)=K_{0}\left[\alpha_{\infty }^{K}+\sum_{i=1}^{N_{K}}\alpha_{i}^{K}e^{-t/\beta_{i}^{K}}\right],$$

where $\alpha_{\infty }$ is the relative weight of the residual element.

The coefficients $\alpha_{i}$ are the relative weights of the relaxation spectrum elements and are dimensionless quantities. To ensure the physical consistency of the model, the normalization condition $\alpha_{\infty }+\sum_{i=1}^{N}\alpha_{i}=1$ is met.

Based on the ratios (2)–(6), the connection with the stress (1) has the form:

(7) $$\sigma \left(t\right)=2G_{0}\int_{0}^{t}\left[\alpha_{\infty }^{G}+\sum_{i=1}^{N_{G}}\alpha_{i}^{G}e^{-t/\beta_{i}^{G}}\right]\frac{de\left(\tau \right)}{d\tau }d\tau +IK_{0}\int_{0}^{t}\left[\alpha_{\infty }^{K}+\sum_{i=1}^{N_{K}}\alpha_{i}^{K}e^{-t/\beta_{i}^{K}}\right]\frac{d\theta \left(\tau \right)}{d\tau }d\tau ,$$

where $e\left(\tau \right)$ is the deviator of the strain tensor and $\theta \left(\tau \right)$ is the volumetric strain.

As part of the work, it is assumed that $K\left(t\right)=K_{0}=\mathrm{const}$. This assumption is typical for a wide class of polymeric materials, which is associated with insignificant effects of relaxation of volumetric deformations. Therefore, the ratio (7) takes the form:

(8) $$\sigma \left(t\right)=2G_{0}\int_{0}^{t}\left[\alpha_{\infty }^{G}+\sum_{i=1}^{N_{G}}\alpha_{i}^{G}e^{-t/\beta_{i}^{G}}\right]\frac{de\left(\tau \right)}{d\tau }d\tau +IK_{0}\theta ,$$

DMA makes it possible to obtain the distribution of Young’s modulus from the frequency and temperature. In a three-point bend, the modulus of elasticity is determined on the basis of the classical Euler–Bernoulli beam theory, in which the deformations are considered small and the shear effects are negligible. Under these conditions, the bending stress-strain state is described by normal stresses associated with deformations through Young’s modulus. Similarly to $G\left(t\right)$, we write the relaxation function for Young’s modulus $E\left(t\right)$:

(9) $$E\left(t\right)=E_{0}\left[c_{\infty }+\sum_{i=1}^{N_{E}}c_{i}e^{-t/\beta_{i}^{E}}\right],$$

where $c_{i}$ is the relative weight of the *i-th* element and $\beta_{i}^{E}$ is the characteristic relaxation time on the *i*-th interval for $E\left(t\right)$.

Assuming the correspondence of the characteristic relaxation times $E\left(t\right)$ and $G\left(t\right)$, the relationship of the relative weights of the functions (5) and (9) can be written as:

(10) $$\sum_{i=1}^{N}\alpha_{i}=\sum_{i=1}^{N}c_{i}\left(E_{0}\left[G_{0}-G_{\infty }\right]/\left(G_{0}\left[E_{0}-E_{\infty }\right]\right)\right)$$

Knowing $K_{0}$, it is possible to determine the initial $G_{0}=3K_{0}E_{0}/\left(9K_{0}-E_{0}\right)$ and final $G_{\infty }=3K_{\infty }E_{\infty }/\left(9K_{\infty }-E_{\infty }\right)$ shear modulus. The modulus of volumetric compression is taken from the experimental data on Poisson’s ratio $K_{0}=E_{0}/\left(3\left(1-2\nu_{0}\right)\right)$.

To take into account the temperature, the WLF temperature–time shift function [54] is used:

(11) $$A_{WLF}\left(T\right)=10^{C_{1}\left(T-T_{r}\right)/\left(C_{2}+\left(T-T_{r}\right)\right)},$$

where $A_{WLF}\left(T\right)$ is the shear function; $T_{r}$ is the base temperature; and $C_{1}$ and $C_{2}$ are material parameters.

Within the framework of this approach, the characteristic relaxation time on the *i*-th interval is associated with (11) ${\;\beta \;}_{i}^{\prime }\left(T\right)=\beta_{i}/A_{WLF}\left(T\right)$. Thus, the temperature’s effect on the behavior of the material is taken into account by changing the characteristic relaxation times, while the relative weights of the elements remain unchanged. This corresponds to the assumption of the thermological simplicity of the material. To describe the storage modulus (12) and the loss modulus (13) obtained using DMA, we will use the following relations:

(12) $$E^{\prime }\left(\omega ;\;T\right)=E_{0}\left[c_{\infty }+\omega^{2}\sum_{i=1}^{N}c_{i}\frac{{{\;\beta \;}_{i}^{\prime }}^{2}}{1+{\left(\omega \;{\;\beta \;}_{i}^{\prime }\right)}^{2}}\right],$$

(13) $$E^{\prime \prime }\left(\omega ;\;T\right)=E_{0}\;\omega \sum_{i=1}^{N}c_{i}\frac{{\;\beta \;}_{i}^{\prime }}{1+{\left(\omega \;{\;\beta \;}_{i}^{\prime }\right)}^{2}},$$

where $E^{\prime }\left(\omega ;\;T\right)$ is the storage modulus; $E^{\prime \prime }\left(\omega ;\;T\right)$ is the loss modulus; and ω is the frequency.

Thus, to describe the thermomechanical behavior of polymer materials, it is necessary to identify the parameters of the Prony series. The vector of unknowns has the form:

(14) $$\bar{x}=\left\{C_{1};\;\;C_{2};\;\;T_{r};\;\;\alpha_{i};\;\;\beta_{i};\;\;i=\bar{1:N}\right\},$$

### 2.4. Identification Procedure

The parameters of the viscoelastic model are determined based on experimental DMA data, in which the frequency and temperature dependencies $E^{\prime }\left(\omega ;\;T\right)$ and $E^{\prime \prime }\left(\omega ;\;T\right)$ are measured.

The identification of the parameters is formulated as a problem of minimizing the deviation between experimental and calculated values. The root-mean-square error acts as a function and has the form:

(15) $$\Phi \left(\bar{x}\right)=\sum_{i=1}^{N_{T}}\left[{\left({E^{\prime }}_{\exp}\left(\omega ;\;T_{i}\right)-{E^{\prime }}_{\mathrm{num}}\left(\omega ;\;T_{i};\;\bar{x}\right)\right)}^{2}+{\left({E^{\prime \prime }}_{\exp}\left(\omega ;\;T_{i}\right)-{E^{\prime \prime }}_{\mathrm{num}}\left(\omega ;\;T_{i};\;\bar{x}\right)\right)}^{2}\right]\to \min$$

Minimization of the functionality $\Phi \left(\bar{x}\right)$ is carried out by the Nelder–Mead method [55] via the gradientless optimization method. It is based on the sequential transformation of the simplex in the parameter space and does not require the calculation of derivatives of the objective function. This makes it effective for solving problems with a nonlinear parametric dependence of the material behavior model.

The physical limitations of the parameters are taken into account in the process of their identification:

(16) $$C_{1}>0;\;\;C_{2}>0;\;\;\alpha_{i}>0,\;\;\beta_{i}>0,\;\;i=\bar{1:N};\;\sum_{i=1}^{N}\alpha_{i}=\left(G_{0}-G_{\infty }\right)/G_{0}.$$

The initial values of the relaxation times are set uniformly on a logarithmic scale, providing coverage of a wide range of characteristic times. The weighting factors are selected based on the normalization condition.

The optimization process is completed when the specified convergence criterion is reached—the error is less than 5%. As a result, the parameters of the Prony series and the temperature shift functions are determined and ensure the best matching of the model with the experimental DMA data.

### 2.5. Numerical Identification Procedure

**Figure 1:**
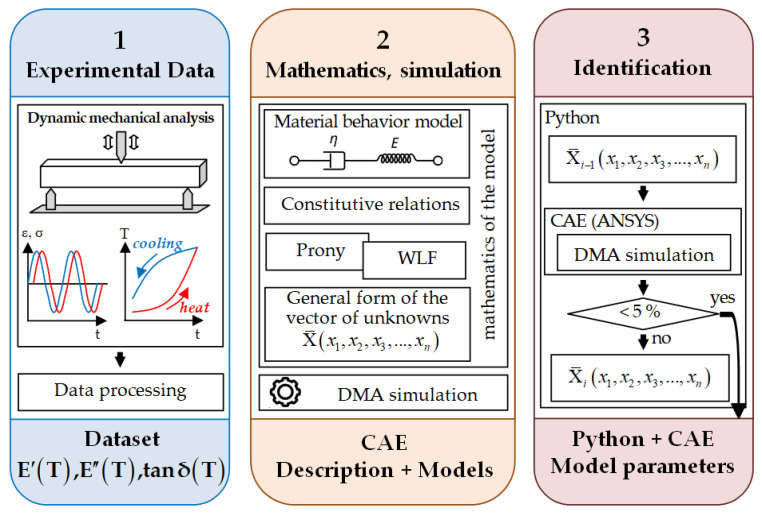
Diagram of a numerical procedure for identifying a mathematical model of the behavior of antifriction materials within the framework of viscoelasticity.

The numerical identification procedure uses the previously described defining ratios and a dataset of experimental studies. Figure 1 shows a diagram of its implementation.

The simplified scheme of the numerical procedure for identifying the material model contains three stages.

The first stage includes the initial processing of data from a series of computational experiments and the formation of a dataset. It includes data on the thermomechanics of materials and is used to evaluate the numerical model.

The second stage includes: the formation of finite element analysis modules, the choice of a material model, and the formation of defining relations, taking into account known theories and the general form of the vector of unknowns. The article considers a viscoelastic model of Maxwell’s body. However, the possibilities of a numerical identification procedure are wider and include the construction of viscoelastic and elastic-visco-plastic models of materials. Simulation modeling of the DMA experiment is also included in the second stage. Computer-Aided Engineering (CAE) modules are the results of the second stage and are used by the model parameter search algorithm.

The numerical algorithm of multiparameter optimization, the formation of the starting vector of unknowns, the management of the implementation of the solution of the problem using CAE software modules, and the automated processing of the results are implemented using Python 3.12. The vector of unknown models is specified iteratively. The numerical identification procedure is completed if the error between the numerical solution and the experimental dataset is less than 5%.

The final vector of the Maxwell model parameters based on Prony series and WLF parameters is formed as a result of the numerical identification procedure. The model is formed in the framework of the script for ANSYS (Livermore, CA, USA).

### 2.6. Analysis of the Behavior of Antifriction Materials During Deformation of a Spherical Support Part, Taking into Account the Temperature Factor

**Figure 2:**
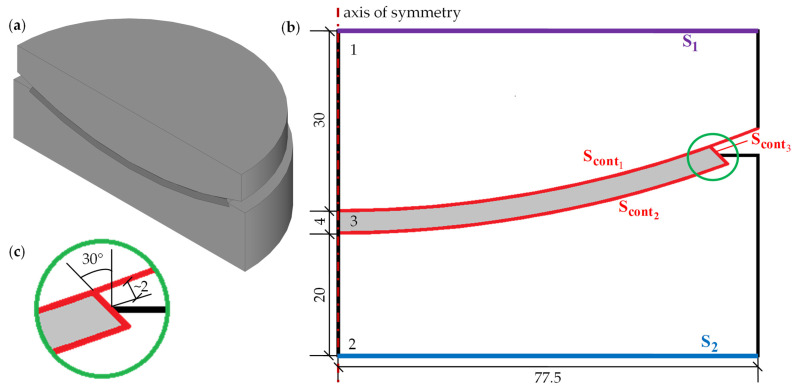
Spherical bridge bearing: (**a**) is the half contact unit (3D image); (**b**) is the computational scheme (central section, axisymmetric design); (**c**) is the end face of the sliding layer; 1 and 2 are top and bottom steel plates, correspondingly; 3 is the polymer/composite sliding layer; the green circle highlights the end face of the sliding layer.

The work of antifriction materials is considered as part of the deformation of the spherical support part L-100 (AlfaTech LLC, Perm, Russia), taking into account the influence of ambient temperature. The computational scheme is presented in Figure 2.

The contact unit of the spherical support part represents two steel plates (1)–(2) separated by an antifriction polymer or composite layer (3). The minimum standard size of the support part designed for the standard vertical load of 1000 kN is considered. The geometric parameters of the structure are shown in Figure 2. The frictional contact is implemented with a previously unknown nature of the distribution of contact states (sliding, adhesion, sliding) $S_{{\mathrm{cont}}_{1}}-S_{{\mathrm{cont}}_{3}}$ at a constant friction coefficient [56]. Vertical movements are prohibited on $S_{2}$. Characteristic load uniformly distributed as pressure (~55 MPa) is applied to $S_{1}$. Bending $S_{1}$ is prohibited. The ambient temperature is considered to be in the range from −40 °C to + 80 °C.

The steel structural elements were modeled in the framework of the theory of elasticity with an elastic modulus of 2e11, a Poisson’s ratio of 0.3, and the CTE of 11.7 × 10^−6^. The friction coefficient is chosen to be a constant value of 0.04 and corresponds to the manufacturer’s data of the bridge bearings for the steel–polymer contact. The friction coefficient value corresponds to the tabulated value for a wide class of antifriction polymer and composite materials. In the first approximation, the Poisson’s ratio of the materials of the sliding layer is 0.466, and the CTE is 8 × 10^−5^; these values are considered to be the same for the entire set of materials. The CTE was selected based on PTFE behavior data. However, it may have a temperature dependence. This requires additional research.

The problem was implemented using the finite element method in ANSYS Mechanical APDL 2021R2 (ANSYS Inc., Livermore, CA, USA). The model is considered in an axisymmetric formulation.

## 3. Results and Discussion

### 3.1. Experiment

**Figure 3:**
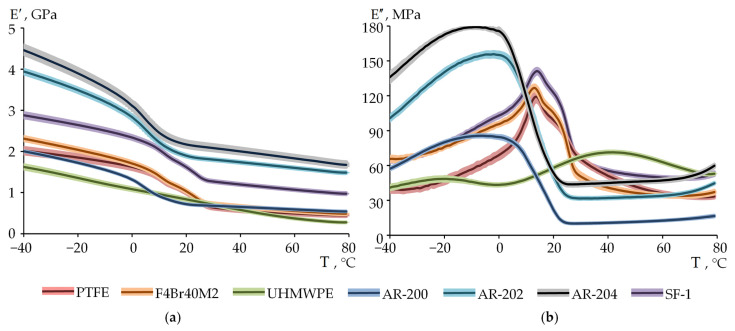
DMA thermogram: (**a**) is the storage modulus; (**b**) is the loss modulus.

**Figure 4:**
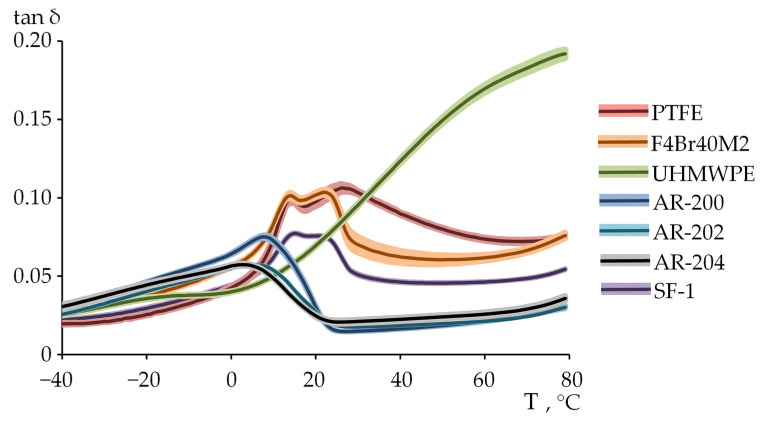
The loss tangent thermogram.

The experimental results are processed and evaluated relative to the standard deviation over the entire temperature range (Figure 3 and Figure 4). The storage and loss modulus thermograms are shown in Figure 3. The loss tangent thermogram is shown in Figure 4.

The maximum average deviation of the storage modulus is observed at a negative ambient temperature and decreases as the temperature rises. The maximum standard deviation of the storage modulus is observed in AR-204: at −40 °C—101.36 MPa; at +80 °C—54.53 MPa. The minimum standard deviation of the storage modulus is observed in AR-200: at −40 °C—5.07 MPa; at +80 °C—4.19 MPa. The standard deviation of the storage modulus of the remaining materials is in the range of 72.27–44.72 MPa at a temperature of −40 °C with a decrease to 23.03–6.56 MPa at +80 °C.

The standard deviation of the loss modulus has lower values compared to the standard deviation of the storage modulus. The nature of the change in the standard deviation of the loss modulus is similar to the storage modulus. The maximum values of the standard deviation of the loss modulus are fixed for PTFE: at −40 °C—5.24 MPa; at +80 °C—1.11 MPa. The minimum standard deviation of the loss modulus is observed for the material AR-200: at −40 °C—1.61 MPa; at +80 °C—0.09 MPa. For the remaining materials, the standard deviation of the loss modulus is 4.43–1.88 MPa at a temperature of −40 °C and decreases to 0.97–0.44 MPa at +80 °C.

The signal of the loss modulus makes it possible to determine the glass transition temperature of the material [57]. The value of the temperature at which the maximum peak $\;E^{\prime \prime }$ is observed shows the glass transition temperature according to ASTME1640-18 “Standard test method for assignment of the glass transition temperature by dynamic mechanical analysis” [58]. No apparent maximum $\;E^{\prime \prime }$ is observed in UHMWPE samples. This means that the glass transition temperature of UHMWPE does not fall within the temperature range in question. The glass transition temperatures of the remaining materials, obtained from the analysis of the temperature dependence $\;E^{\prime \prime }\left(T\right)$, are: +14 °C (288.15 K) is PTFE; +13 °C (287.15 K) is F4Br40M2; −6 °C (268.15 K) is AR-200; −2 °C (272.15 K) is AR-202; −9 °C (265.15 K) is AR-204; and +14 °C (288.15 K) is SF-1. This is confirmed by the loss tangent thermogram.

The introduction of the nanofiller into the PTFE matrix leads to an increase in the storage modulus. The maximum effect is observed with modified PTFE filled with carbon fiber (AR-204). A similar trend is observed when carbon fiber is introduced as a nano-additive into polymer materials of a different nature and manufacturing technology [59]. The casting coke spiked material (AR-202) also has a significant increase in the storage modulus compared to pure PTFE. In the materials AR-202 and AR-204, modified PTFE is used in the matrix composition. It is assumed that the combined effect of radiation exposure of the matrix and the nano-additive has the maximum effect of increasing the thermomechanical parameter. This is confirmed by the fact that a separate modification of PTFE (AR-200) and the introduction of a carbon fiber nano-additive (SF-1) did not allow such an effect. The temperature dependence distribution of the storage modulus is generally similar for all materials.

The effect of PTFE modification on the behavior of materials can be assessed using the loss modulus. The inclusion of nano-additives in the PTFE matrix leads to an increase in the loss modulus compared to pure PTFE, but does not affect the temperature dependence of the parameter. Modification of PTFE leads to a change in the dependence $\;E^{\prime \prime }\left(T\right)$, a shift in the glass transition temperature to the temperature range below 0 °C, and a change in the rate of relaxation transition of materials. The materials have a transition region from a vitrified to a highly elastic state near the glass transition temperature. High moduli of losses of composites and PTFE modifications at negative temperatures enhance viscoelasticity and provide greater energy dissipation [60]. At positive temperatures, this trend is observed only in SF-1, while the differences from pure PTFE do not exceed 30 MPa.

The behavior of UHMWPE is significantly different from the behavior of PTFE, its modifications, and compositions, and is considered one of its alternatives. This is largely due to the wide distribution of the material in the polymer market [61] as well as its use as a sliding layer [48,62]. Areas of relaxation transitions are not observed in the temperature range under consideration. The material exhibits a greater elastic response than PTFE at a temperature range of [−4; +30] °C. Two temperature zones are observed where the material exhibits higher viscosity than PTFE.

### 3.2. Thermoviscoelastic Model of the Behavior of an Antifriction Material

#### 3.2.1. Numerical Analog of the Material

Prony series description parameters were obtained for each material. The total number of iterations did not exceed 3000 when identifying parameters, with an average calculation time of about 10 min.

**Figure 5:**
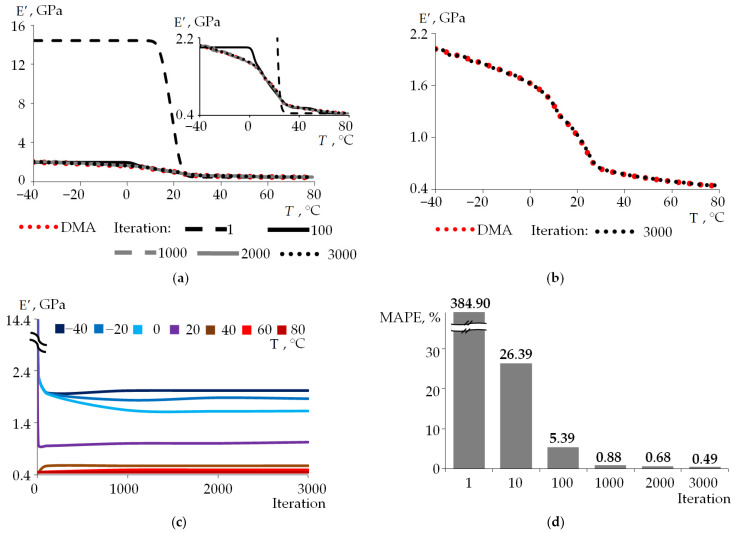
The convergence of the algorithm for identifying model parameters: (**a**) is the convergence at different iterations; (**b**) is a comparison of experimental data and solutions for the optimal vector of unknowns; (**c**) is the dependence of the value of the storage modulus on iterations at different temperatures; (**d**) is the mean absolute percentage error (MAPE).

Figure 5 shows an example of the convergence of the parameter identification algorithm for $E^{\prime }$ with different iterations (1, 100, 1000, 2000, and 3000) for PTFE material.

The initial values of the parameters lead to a significant error between the experimental and calculated data (root of the mean square error RMSE = 8.749). After 100 iterations, there is a satisfactory agreement of the results at temperatures below the glass transition temperature. The relative value of mean absolute percentage errors (MAPE) is on average not more than 6%, RMSE = 0.117.

The calculated values differ significantly from the experimental ones at temperatures above the glass transition temperature, which indicates a more complex nature of relaxation processes in this range.

The results obtained at 1000 and 2000 iterations demonstrate a marked improvement in approximation. However, the algorithm does not recognize them as the optimal solution. Fluctuations of $E^{\prime }$ are observed, although its values coincide well with the experimental data. The model parameters should provide a discrepancy between the numerical solution and the experiment of less than 5% over the entire data set. With an increase in the number of iterations to 3000, a good match of the model with the experimental data is achieved (MAPE = 0.49%, RMSE = 0.007). In this case, the solution is optimal. Deviations of numerical analogs from experimental data in the analysis of other materials have small differences from the deviations obtained for PTFE.

Based on the results of this procedure, parameters were obtained to describe the viscoelastic behavior of materials using Maxwell type equations (Table 3 and Table 4), as well as WLF parameters (Table 3).

In the first approximation, one set of parameters is used to describe the behavior of materials in a temperature range from −40 °C to +80 °C. However, in almost all materials (except UHMWPE), the glass transition temperature falls into the considered temperature range. There is a hypothesis that it is necessary to take into account the differences in the behavior of materials before and after reaching the glass transition temperature [63,64]. Temperature has a strong impact on the behavior of polymer and composite materials [65]. The glass transition temperature is one of the key factors determining the viscoelastic behavior of polymer materials [66]. The main limitation of the current model of thermomechanics of antifriction materials is one set of parameters to describe their behavior over the entire temperature range of operation. The direction of future research is the expansion of the identification procedure and modification of the algorithm for modeling the behavior of materials, taking into account the difference in the nature of their mechanical response at temperatures below and above the glass transition temperature.

#### 3.2.2. Analysis of the Influence of Finite-Element Partitioning on the Numerical Solution of the Problem

The first stage of the numerical study included an assessment of the effect of the finite element (FE) mesh on the numerical solution of the problem. Repeatability was investigated at an ambient temperature of +20 °C. The material of the sliding layer was PTFE. Earlier studies found the presence of fluctuations $S_{{\mathrm{cont}}_{1}}$ due to the lack of complete coincidence of the nodes of the mesh of CONTA-TARGE elements. TARGE $S_{{\mathrm{cont}}_{1}}$ and CONTA $S_{{\mathrm{cont}}_{2}}$ were separated in such a way as to ensure the maximum overlap at initial contact. The formation of the FE mesh on the interface surfaces of elements has a strong impact on the solution of contact problems, despite the development of computing packages, methods, approaches, and algorithms [67,68]. Conformal models require the formation of a node-to-node mesh to eliminate the constraints of solving the problem.

**Figure 6:**
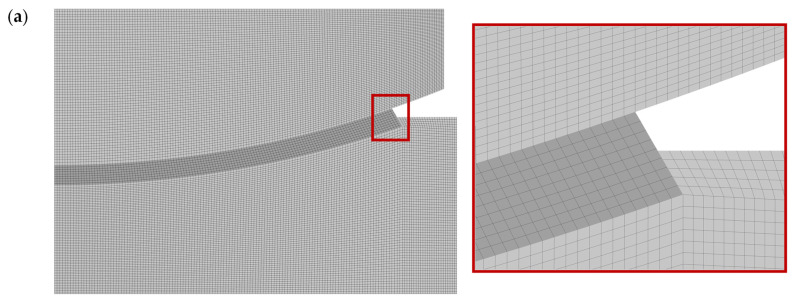

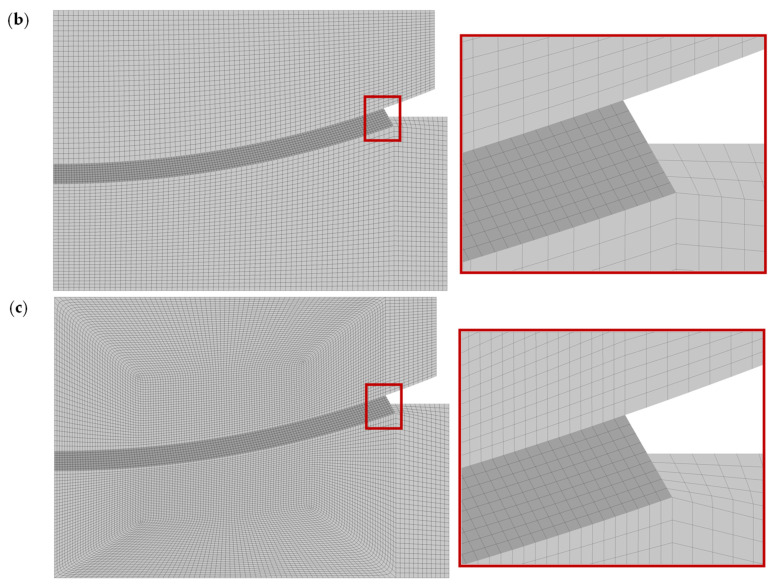
Finite element partition: (**a**) is mesh 1; (**b**) is mesh 2; (**c**) is mesh 3.

Three variants of the FE mesh were considered: 1 is a uniform mesh with the same overall size of elements in all volumes of the model; 2 is a mesh in which the overall size in steel elements is twice as large as in the sliding layer; 3 is a mesh with a uniform partition in the volume of the sliding layer and a gradient increase in the size of the element in steel plates from the contact area (the maximum size of the elements was doubled to the surfaces remote from the contact zone). All meshes are implemented using quadrilateral elements with Lagrangian approximation. Figure 6 shows the view of FE meshes for the sliding layer with the overall size of the elements set to 0.5 mm.

Two types of finite elements are considered for constructing the FE mesh: 1 is first-order elements (PLANE182, CONTA171, TARGE169); 2 is second-order elements (PLANE183, CONTA172, TARGE169). Higher-order elements have been considered in connection with their effectiveness for modeling nonlinear contact, especially in the nonlinear behavior of materials [69].

Past studies have established the need for high-quality construction of an FE mesh in the area of mating of steel elements and a sliding layer [70]. The minimum overall dimension of the element is related to the thickness of the sliding layer. Table 5 presents the FE mesh parameters.

**Figure 7:**
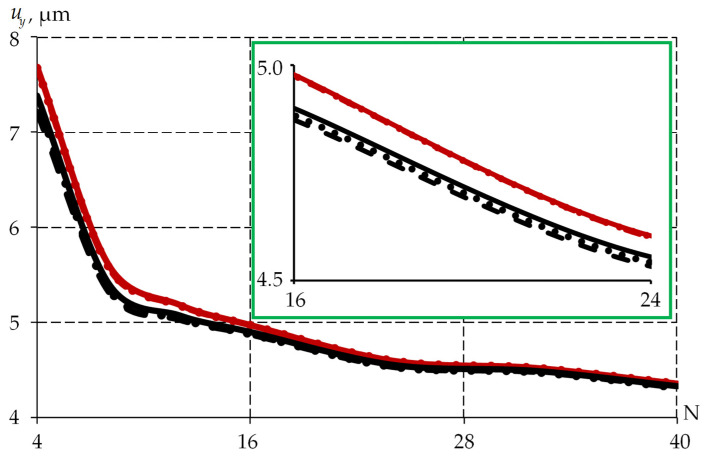
The convergence of the numerical solution of the problem by the example of vertical displacements: black is elements of the first order; red is elements of the second order; the solid line is mesh 1; the stroke is mesh 2; points are mesh 3.

The article investigated the convergence of the numerical solution through the example of maximum vertical displacements (Figure 7).

The change in the values of the vertical displacement of the support part does not exceed 8% for all types of mesh elements, when dividing the sliding layer into 16 and 24 elements. Further refinement of the mesh leads to a change in the parameter by no more than 1.2–3.6%. It can be noted that the value of the parameter is higher when using two order elements. However, the differences do not exceed 5% in the case of meshes 1 and 3 and 6.3% in the case of mesh 2. The difference in the vertical displacements of the model does not exceed 2% when using elements of the 1st and 2nd order with a mesh with 16 elements along the thickness of the layer.

For all mesh configurations, the maximum values of stress intensity and contact pressure differ by less than 1% for meshes with division into 16 and 24 elements by the thickness of the sliding layer.

Differences in solutions with different meshes with first-order elements do not exceed 2.5%, with second-order elements less than 0.6%. However, when using first-order elements in mesh 2, fluctuations $S_{{\mathrm{cont}}_{1}}$ are observed—local variations in the contact pressure in adjacent elements in the range of about 2 MPa.

The time for solving the problem within the framework of static loading is several seconds when using first-order elements with a gradient increase in the element from the interface area. Mesh 2 converges more slowly than the other two FE meshes. The use of higher-order elements also increases the calculation time, and their use is not always justified and effective. At the same time, elements with linear functions of the shape make it possible to obtain a high-quality solution with qualitatively constructed meshes and properly developed algorithms [71,72].

As a rational mesh, a mesh was chosen using first-order elements, containing 16 elements in the thickness of the interlayer and a gradient increase in the elements in the steel plates as the distance from the contact surfaces with the sliding layer. The discretization of the system is approximately 116 thousand nodal unknowns in such a partition. It provides the required accuracy of the solution without increasing the load of computing power. This will be relevant when moving to the analysis of the influence of the time factor and the frequency of loading, which are planned in future studies.

#### 3.2.3. Thermomechanical Deformation of the Spherical Support Part of the Bridge

**Figure 8:**
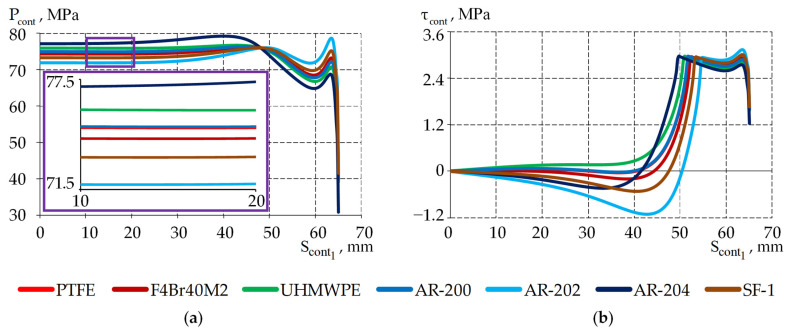
Contact parameters $S_{{\mathrm{cont}}_{1}}$: (**a**) is the contact pressure; (**b**) is the contact shear stress.

The influence of temperature on the behavior of the materials of the sliding layer under the deformation of the spherical support part under the influence of the nominal load is analyzed. Figure 8 shows, by way of example, the distribution of contact parameters at an ambient temperature of +20 °C. The contact parameters are shown for $S_{{\mathrm{cont}}_{1}}$, along which it is possible to rotate the spherical segment.

The nature of the distribution of the contact pressure and the contact shear stress at $S_{{\mathrm{cont}}_{1}}$ does not depend on the material of the sliding layer. Divergence of the contact surfaces near the edge of the sliding layer is not observed for this temperature.

Modification of PTFE has no significant effect on the contact characteristics. The contact parameters of PTFE and AR-200 have small differences: contact pressure is less than 1%; contact shear stress is no more than 3% (on the main contact area). The differences can reach 36% near the zone of change of contact states from sliding to adhesion.

The stick zone under full stick conditions is observed in the central part of the sliding surface. The maximum surface area in the adhesion state is observed in a structure with a layer of AR-202 (58.63% of the total contact area). The minimum is observed in a structure with a UHMWPE interlayer (47.41%). The boundary of the stick zone under full stick conditions can be determined by changing the contact parameters. The contact pressure varies slightly in the adhesion area (central zone of the support part). The zone in which the contact shear stress reaches the plateau (stabilizes) corresponds to the transition from adhesion to sliding. The radius of the area of full adhesion of the interface surfaces $S_{{\mathrm{cont}}_{1}}$ varies from 49.39 to 54.55 mm.

The maximum level of contact pressure values during viscoelastic setting is offset relative to the center of the support part. It is observed mainly near the boundary of the transition of the contact state from adhesion to sliding. The maximum level of contact parameters is observed near the edge of the sliding layer of AR-202. Thus, for the contact pressure, it reaches 9.34% compared to the central part of the structure. The minimum increase in contact pressure relative to the central part (1.04%) is noted in a structure with a UHMWPE layer.

Basically, the maximum level of contact parameters of modern materials is commensurate with the interlayer of pure PTFE. In the comparative analysis with pure PTFE, the maximum differences in contact pressure were recorded for the layers of AR-202 (2.8%) and AR-204 (3.7%), while the maximum difference in contact shear stress (6.04%) was observed for the layer of AR-202. The contact pressure in the center of the supporting part of the structure with a layer of AR-202 is 4.17% less, and that of AR-204 is 2.88% more than in the structure with a PTFE interlayer. The difference of the remaining materials from the PTFE interlayer in contact parameters is less than 1%.

The absolute values of the contact parameters at $S_{{\mathrm{cont}}_{2}}$ have small differences from $S_{{\mathrm{cont}}_{1}}$. The nature of the distribution of the contact pressure is more uniform, which is associated with the limited freedom of the interface. The nature of the distribution of the contact shear stress is opposite.

**Figure 9:**
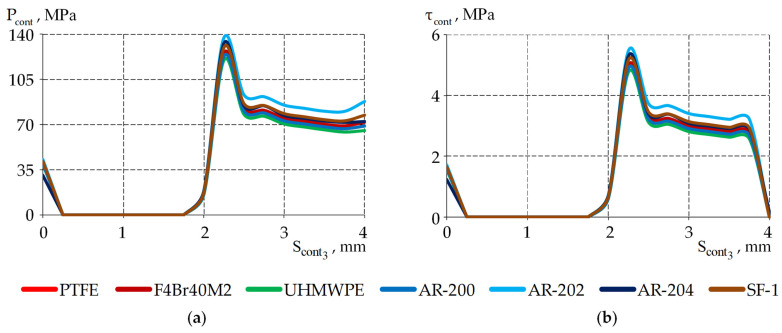
Contact parameters $S_{{\mathrm{cont}}_{3}}$: (**a**) is the contact pressure; (**b**) is the contact shear stress.

The maximum level of contact parameters is observed at $S_{{\mathrm{cont}}_{3}}$ (Figure 9). This is largely due to the relative freedom of the end of the sliding layer $S_{{\mathrm{cont}}_{3}}$ as well as the presence of a stress concentrator in the area of the steel collar of the bottom plate of the support part.

The maximum level of contact parameters is observed near the edge of the mating of the end of the sliding layer with the lower steel plate. The maximum contact pressure level is on average 64.85% higher compared to $S_{{\mathrm{cont}}_{1}}-S_{{\mathrm{cont}}_{2}}$. The maximum level of the contact shear stress is on average 69.54% higher. The minimum values of the parameters are observed in a structure with a layer of UHMWPE. The maximum values are observed in a structure with a layer of AR-202. All PTFE composites have a higher level of contact parameters than a pure PTFE interlayer at $S_{{\mathrm{cont}}_{3}}$.

**Figure 10:**
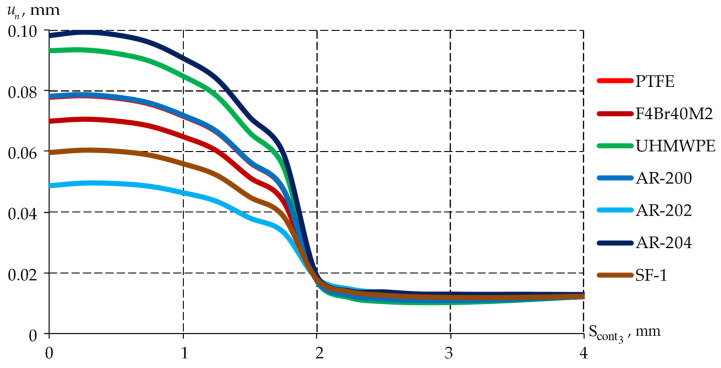
Movements along the normal of the end of the sliding layer.

The displacements along the normal of the end of the sliding layer are one of the indicators of the material’s work as part of the deformation of the spherical support part (Figure 10).

The maximum normal displacements of the end of the sliding layer do not exceed 0.1 mm and are observed in the AR-204 layer. The minimum normal displacements are observed in the layer of AR-202 and reach 0.049 mm. The maximum normal displacements of the sliding layers of composite materials F4Br40M2 and SF-1 (0.07 and 0.06 mm, respectively) are less than that of a pure PTFE interlayer.

Temperature dependencies are obtained for the parameters of the contact and the stress-strain state of the sliding layer. The temperature dependencies of the maximum values of the parameters are well described by polynomial regression. The polynomial of the second degree well describes the behavior of the volume of the sliding layer and the main contact surfaces $S_{{\mathrm{cont}}_{1}}-S_{{\mathrm{cont}}_{2}}$. The dependence of the contact parameters of the end of the sliding layer is described by linear regression. The temperature dependencies of the main contact parameters of the stress-strain state are presented in Appendix A.

**Figure 11:**
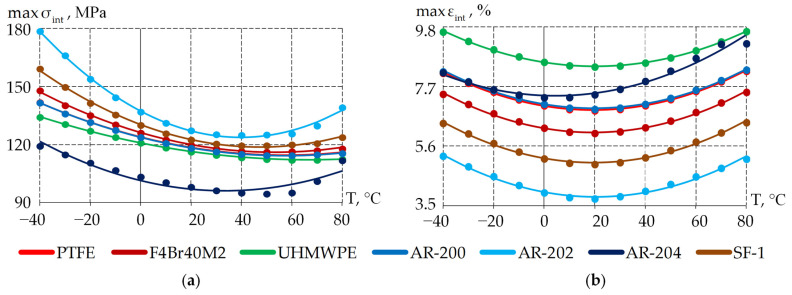
The temperature dependence of the maximum level of the stress-strain state parameters: (**a**) is the contact pressure; (**b**) is the contact shear stress; the marker is the numerical data; the line is the approximation.

Figure 11 shows the temperature dependencies of the maximum intensity of stresses and deformations.

The maximum stress intensity is more sensitive to ambient temperature. The value of the parameter is higher at negative temperatures.

The maximum stress intensity on average is described with a coefficient of determination R^2^ of 0.9856 and an average error size RMSE of 0.1848. Approximately 50% of the volume of the sliding layer experiences hydrostatic compression due to the presence of a full adhesion zone in the contact. The maximum stress intensity is observed near the stress concentrator of the end of the sliding layer. This applies to the structural features of the support part and does not depend on the material model [48,73]. The material model and external factors, including temperature, affect the maximum values of the parameter and the volume of the layer in which the maximum is observed. The location of the maximum stresses of the spherical sliding layer is correlated with the data of Wei et al. [74].

The maximum stress intensity in the volume of the sliding layer of F4Br40M2; UHMWPE differs from PTFE on average by no more than 3%. In a structure with a layer of SF-1, this figure is on average 6% higher. The maximum differences in stress intensity are observed in the layers of AR-202 and AR-204. The stress intensity of the AR-202 layer is on average 13% higher. The opposite effect is observed in the AR-204 layer, where the stress intensity is on average 15% less. The minimum differences in stress intensity compared to pure PTFE are observed in the AR-200 layer and reach a maximum of 0.13%.

The maximum intensity of deformations has a greater spread between the materials of the sliding layer. The average coefficient of determination R^2^ of the mathematical description of the temperature dependence of the maximum strain intensity is 0.99 at RMSE 0.0131. The maximum intensity of deformations of the sliding layer does not exceed 10%.

A comparative analysis of the maximum intensity of deformations in a layer of polymer/composite materials with a PTFE layer was carried out. For AR-202, the parameter values are on average 41% less. For UHMWPE, the maximum strain intensity is on average 20% higher. Minimal differences in the parameter are observed in AR-200 (less than 0.65%).

**Figure 12:**
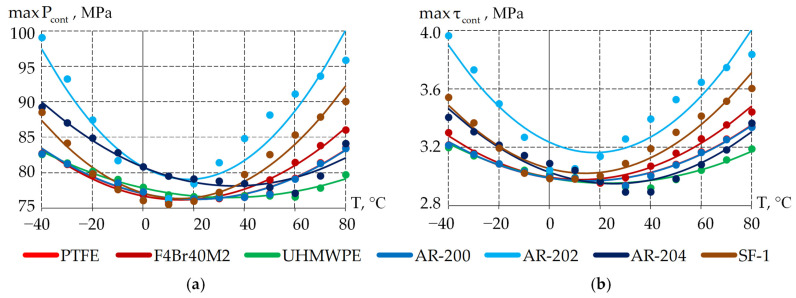
The temperature dependence of the maximum level of the contact parameters $S_{{\mathrm{cont}}_{1}}$ on T: (**a**) is the contact pressure; (**b**) is the contact shear stress; the marker is the numerical data; the line is the approximation.

Figure 12 shows the temperature dependence of the maximum contact parameters on the main interface $S_{{\mathrm{cont}}_{1}}$.

The minimum values of the contact parameters are observed at a temperature range of [0; +20] °C. A comparable level of contact parameters is observed at maximum negative and positive temperatures. AR-202 has a maximum value of contact parameters and is more sensitive to ambient temperature compared to other materials. A higher-order polynomial can better describe the temperature dependence of contact parameters for the AR-200. The second-order polynomial describes the contact pressure with the lowest coefficient of determination R^2^ = 0.8732 with the highest value of RMSE = 0.7199.

The material of the sliding layer significantly affects the contact parameters. The maximum deviations relative to the PTFE interlayer are observed for AR-202: the contact pressure is on average 9.67% higher, and the contact shear stress is 11.88% higher. For UHMWPE, the deviation of the maximum contact pressure is on average 0.67% less, and the contact shear stress is 1.47% less. The use of other materials within the sliding layer leads to a slight increase in contact parameters, on average by no more than 4%.

Physical determination of the level of contact parameters is a difficult task. This is largely due to the geometric configuration of the structure. Therefore, numerical modeling to determine the contact parameters is common [75,76]. Recently, however, there has been a tendency to create intelligent bearings with the introduction of sensors into the design [77,78,79]. While this type of monitoring is not aimed at determining the contact characteristics of the system, it can be assumed that the further digitalization of the bearing construction industry over time will make it possible to determine the contact parameters when monitoring the state of the structure.

**Figure 13:**
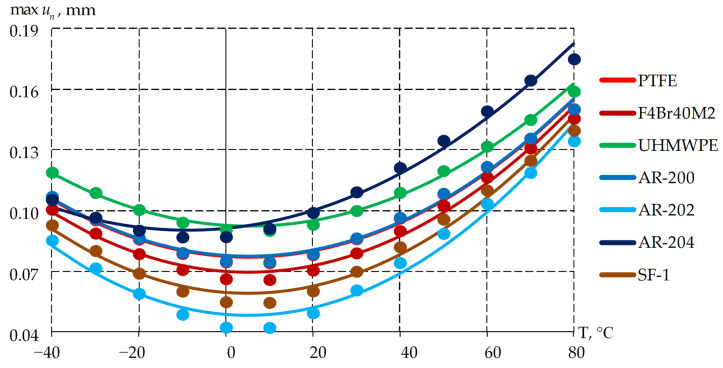
The temperature dependence of the maximum normal movements of the sliding layer end: the marker is the numerical data; the line is the approximation.

Figure 13 represents the temperature dependence of the maximum normal movements of the end of the sliding layer.

The maximum normal displacements of the end of the sliding layer do not exceed 0.2 mm and are observed at an ambient temperature of +80 °C. For AR-202, this parameter is lower by an average of 26% compared to PTFE. A similar comparison shows that for AR-204, on average, normal displacements are 16.9% greater.

The level of displacements of the spherical bearing is commensurate with the results of experimental studies presented by Chen et al. [80].

Spherical bridge bearings are among the three most common configurations used for the mobility of the bridge span and damping loads from external influences [81]. Common defects of spherical bearings of bridges are destruction, strong deformation of Teflon layers of sliding, as well as degradation of the material. This confirms the need to assess the suitability of modern materials with improved physical and mechanical properties for use in antifriction sliding layers. Masi et al. emphasize the need to form a database on the reaction of materials to different types of load and temperature. Other authors [82,83] also emphasize the importance of assessing the effect of temperature effects on the operation of bridge bearings. Thus, temperature accounting is necessary to describe the behavior of non-metallic bearing materials. Within the framework of the current study, this is realized by constructing thermoviscoelastic models of the behavior of the materials used or suitable for the sliding layers. This approach makes it possible to assess the behavior of the structure, taking into account the temperature and inelastic effects, which is important for the rationalization of its work [84].

## 4. Conclusions

It should be noted that the results were obtained within the framework of several assumptions adopted in constructing the numerical model. Specifically, the friction coefficient (μ = 0.04) and Poisson’s ratio (ν = 0.466) were assumed constant over the entire temperature range under consideration. A single set of Prony series parameters was used to describe the thermoviscoelastic behavior of each material in the temperature range from −40 °C to +80 °C. However, the physical and mechanical properties of polymeric materials can vary significantly near the glass transition temperature. The adopted assumptions can affect the absolute values of stresses, displacements, and contact pressures. Despite its limitations, the model provides a valid comparative study of a set of materials. The model can be used for a preliminary assessment of the effectiveness of materials as antifriction layers in bridge bearings.

The thermomechanics of a set of modern nanomodified polymers and composites suitable for use as sliding layers of friction units have been studied. The temperature dependencies of viscoelastic characteristics of materials have been determined at an ambient temperature of −40 to +80 °C. Numerical analogs of materials have been constructed according to experimental data (MAPE < 0.5%, RMSE < 0.01).

The behavior of these materials as a sliding layer of the spherical support part in the entire range of operating temperatures has been investigated.

The nonlinear temperature dependence of the parameters of the contact stress-strain state is observed during deformation of the materials of the spherical support part. Mathematical temperature dependencies of the parameters of the stress-strain state and contact are established. They are suitable for analyzing the behavior of the structure over the operating temperature range. Mathematical dependencies have an average coefficient of determination R^2^ = 0.97 and an average error size RMSE = 0.092.

AR-204 and SF-1 are promising alternatives to PTFE for molding the sliding layer.

The sliding layer from AR-204 makes it possible to obtain an improvement in the stress-strain state over the entire range of operating temperatures. The stress intensity is on average 15% less. The increase in the intensity of deformations is insignificant (less than 2%). However, at positive temperatures, the material has a greater level of movement of the end of the sliding layer than that of the Teflon sliding layer. In a similar comparison, the contact pressure is on average 3.22% higher.

SF-1 shows, on average, an increase in stresses of less than 7% with a decrease in deformations of about 2% compared to PTFE. Contact parameters are more than PTFE parameters (on average by 3–4%). However, the sliding layer has less end deformation over the entire range of operating temperatures. The maximum normal displacements of the end of the sliding layer are on average 16.6% less than that of the PTFE sliding layer.

As a recommendation, it can be noted that AR-204 is the most promising material for use in low-temperature conditions due to its high stiffness and stable mechanical properties. AR-202 and SF-1 materials can be considered effective alternatives to traditional PTFE in moderate operating temperatures due to their combination of increased stiffness and energy dissipation capacity.

Current studies do not provide a 100% guarantee of the correct choice of rational materials of the sliding layer. This is due to the limitations of the model. Data on the temperature dependence of thermal expansion coefficients are necessary to clarify the model. Analysis of the behavior of materials during wear and aging is required to take them into account in the numerical model.

## Figures and Tables

**Table 1:** The main properties of the materials under consideration.

Material	Density, g/cm^3^	Brinell Hardness, N/mm^2^	Modulus of Elasticity, MPa
PTFE	2.159–2.169	30.2–33.1	545
F4Br40M2	3.050–3.120	35.7–39.9	903
UHMWPE	-	39.1–41.8	706
AR-200	2.195–2.215	40–42	864
AR-202	2.195–2.215	40–42	880
AR-204	2.190–2.210	55–58	1000–1100
SF-1	1.960–2.030	65–78	750–1000

**Table 2:** The parameters near the exit zone from the linear theory of viscoelasticity.

Material	PTFE	F4Br40M2	UHMWPE	AR-200	AR-202	AR-204	SF-1
Amplitude, µm	40	40	150	175	100	75	45
Strain, %	0.0275	0.0275	0.1031	0.1203	0.0688	0.0516	0.0309

**Table 3:** The parameters of the Prony series and WLF.

Material	$C_{1}$	$C_{2}$	$T_{r}$, °K	$E_{0}$, MPa	$E_{\infty }$, MPa
PTFE	203.908	849.003	270.214	2021.386	446.325
F4Br40M2	299.100	1069.367	263.790	2313.682	489.782
UHMWPE	288.378	1393.953	274.120	1624.766	280.550
AR-200	103.243	419.182	266.802	2006.566	541.677
AR-202	128.304	438.184	260.565	3945.537	1486.422
AR-204	51.796	175.616	253.551	4467.330	1675.070
SF-1	191.149	781.408	267.538	2882.213	974.841

**Table 4:** The 40 $\alpha_{i}-\beta_{i}$ pairs.

i	$\beta_{i}$	PTFE	F4Br40M2	UHMWPE	AR-200	AR-202	AR-204	SF-1
		$\alpha_{i}$	$\alpha_{i}$	$\alpha_{i}$	$\alpha_{i}$	$\alpha_{i}$	$\alpha_{i}$	$\alpha_{i}$
1	1.00 × 10^−9^	3.04 × 10^−2^	2.42 × 10^−2^	3.08 × 10^−2^	2.90 × 10^−2^	1.12 × 10^−2^	5.13 × 10^−3^	2.39 × 10^−2^
2	5.54 × 10^−9^	2.54 × 10^−6^	1.12 × 10^−2^	3.14 × 10^−2^	1.28 × 10^−2^	1.33 × 10^−2^	1.15 × 10^−3^	1.49 × 10^−8^
3	3.07 × 10^−8^	2.14 × 10^−2^	1.22 × 10^−2^	2.41 × 10^−2^	2.13 × 10^−2^	1.08 × 10^−2^	2.40 × 10^−4^	1.48 × 10^−2^
4	1.70 × 10^−7^	1.40 × 10^−2^	1.83 × 10^−2^	2.68 × 10^−2^	2.57 × 10^−2^	1.39 × 10^−2^	1.24 × 10^−2^	8.64 × 10^−3^
5	9.43 × 10^−7^	4.73 × 10^−9^	1.23 × 10^−2^	3.26 × 10^−2^	5.22 × 10^−5^	2.03 × 10^−2^	3.01 × 10^−3^	1.53 × 10^−2^
6	5.22 × 10^−6^	2.17 × 10^−2^	1.70 × 10^−2^	3.86 × 10^−2^	4.47 × 10^−2^	5.17 × 10^−9^	2.50 × 10^−2^	1.00 × 10^−2^
7	2.89 × 10^−5^	1.76 × 10^−2^	2.28 × 10^−2^	2.58 × 10^−2^	4.84 × 10^−3^	2.78 × 10^−2^	2.49 × 10^−2^	2.12 × 10^−2^
8	1.60 × 10^−4^	1.85 × 10^−2^	2.93 × 10^−12^	3.68 × 10^−2^	3.17 × 10^−2^	8.19 × 10^−3^	1.33 × 10^−3^	1.11 × 10^−5^
9	8.89 × 10^−4^	2.38 × 10^−8^	2.94 × 10^−2^	2.55 × 10^−2^	2.17 × 10^−2^	1.80 × 10^−2^	1.48 × 10^−2^	2.10 × 10^−2^
10	4.92 × 10^−3^	2.02 × 10^−2^	2.35 × 10^−2^	2.16 × 10^−2^	2.34 × 10^−2^	1.98 × 10^−2^	4.86 × 10^−3^	2.13 × 10^−2^
11	2.73 × 10^−2^	1.83 × 10^−2^	7.84 × 10^−9^	3.75 × 10^−2^	4.75 × 10^−2^	1.33 × 10^−2^	3.10 × 10^−2^	7.11 × 10^−12^
12	1.51 × 10^−1^	2.83 × 10^−2^	2.57 × 10^−2^	2.53 × 10^−2^	3.14 × 10^−5^	2.11 × 10^−2^	1.59 × 10^−2^	3.78 × 10^−2^
13	8.38 × 10^−1^	1.83 × 10^−2^	2.69 × 10^−2^	2.73 × 10^−2^	6.33 × 10^−2^	2.23 × 10^−2^	2.34 × 10^−2^	3.00 × 10^−10^
14	4.64 × 10^0^	2.85 × 10^−2^	1.40 × 10^−2^	3.15 × 10^−2^	2.66 × 10^−2^	1.38 × 10^−2^	2.02 × 10^−2^	4.46 × 10^−2^
15	2.57 × 10^1^	3.66 × 10^−2^	2.32 × 10^−2^	2.11 × 10^−2^	1.06 × 10^−1^	4.59 × 10^−2^	1.83 × 10^−2^	1.10 × 10^−7^
16	1.43 × 10^2^	9.30 × 10^−2^	2.34 × 10^−2^	2.05 × 10^−2^	3.88 × 10^−2^	2.39 × 10^−15^	1.94 × 10^−2^	5.58 × 10^−2^
17	7.90 × 10^2^	3.94 × 10^−2^	5.56 × 10^−2^	4.37 × 10^−2^	7.59 × 10^−2^	5.49 × 10^−2^	2.69 × 10^−2^	4.15 × 10^−2^
18	4.38 × 10^3^	5.13 × 10^−2^	1.12 × 10^−15^	4.63 × 10^−2^	2.91 × 10^−2^	3.80 × 10^−2^	4.77 × 10^−2^	4.73 × 10^−2^
19	2.42 × 10^4^	5.72 × 10^−2^	8.37 × 10^−2^	3.79 × 10^−5^	3.20 × 10^−2^	5.11 × 10^−2^	3.86 × 10^−2^	6.10 × 10^−2^
20	1.34 × 10^5^	8.20 × 10^−2^	4.10 × 10^−2^	2.59 × 10^−2^	1.38 × 10^−2^	4.13 × 10^−2^	3.51 × 10^−2^	2.63 × 10^−2^
21	7.44 × 10^5^	8.56 × 10^−2^	4.19 × 10^−2^	6.33 × 10^−2^	1.58 × 10^−2^	3.36 × 10^−2^	6.18 × 10^−2^	7.91 × 10^−2^
22	4.12 × 10^6^	1.24 × 10^−2^	3.40 × 10^−2^	6.45 × 10^−6^	3.35 × 10^−3^	2.26 × 10^−2^	4.38 × 10^−2^	2.42 × 10^−2^
23	2.29 × 10^7^	1.20 × 10^−2^	5.52 × 10^−2^	3.32 × 10^−2^	2.99 × 10^−3^	1.86 × 10^−2^	2.22 × 10^−2^	1.51 × 10^−2^
24	1.27 × 10^8^	1.22 × 10^−2^	5.91 × 10^−2^	3.20 × 10^−2^	4.13 × 10^−3^	1.99 × 10^−2^	2.93 × 10^−2^	1.70 × 10^−2^
25	7.02 × 10^8^	6.11 × 10^−10^	3.79 × 10^−2^	2.66 × 10^−2^	8.45 × 10^−5^	1.94 × 10^−13^	3.48 × 10^−3^	3.64 × 10^−6^
26	3.89 × 10^9^	1.66 × 10^−2^	2.40 × 10^−15^	1.51 × 10^−2^	1.01 × 10^−2^	5.37 × 10^−3^	5.87 × 10^−3^	1.48 × 10^−2^
27	2.15 × 10^10^	4.07 × 10^−3^	1.59 × 10^−2^	2.56 × 10^−2^	8.59 × 10^−3^	1.51 × 10^−2^	3.32 × 10^−3^	6.91 × 10^−8^
28	1.19 × 10^11^	5.14 × 10^−5^	1.53 × 10^−3^	2.83 × 10^−2^	7.32 × 10^−5^	3.60 × 10^−14^	1.15 × 10^−2^	4.65 × 10^−3^
29	6.61 × 10^11^	1.97 × 10^−2^	5.44 × 10^−3^	1.21 × 10^−2^	1.24 × 10^−2^	4.69 × 10^−11^	3.35 × 10^−3^	1.79 × 10^−2^
30	3.67 × 10^12^	1.48 × 10^−6^	1.62 × 10^−2^	1.13 × 10^−2^	7.92 × 10^−3^	1.17 × 10^−11^	1.30 × 10^−2^	1.11 × 10^−9^
31	2.03 × 10^13^	1.33 × 10^−2^	5.48 × 10^−8^	2.79 × 10^−3^	1.44 × 10^−3^	1.64 × 10^−2^	1.51 × 10^−2^	1.04 × 10^−2^
32	1.13 × 10^14^	5.21 × 10^−7^	1.30 × 10^−2^	1.20 × 10^−3^	6.84 × 10^−3^	5.19 × 10^−3^	3.81 × 10^−4^	1.27 × 10^−2^
33	6.24 × 10^14^	1.45 × 10^−4^	1.43 × 10^−12^	8.18 × 10^−3^	5.81 × 10^−3^	4.54 × 10^−14^	9.57 × 10^−3^	1.41 × 10^−5^
34	3.46 × 10^15^	9.02 × 10^−8^	8.15 × 10^−3^	8.18 × 10^−8^	4.67 × 10^−3^	1.10 × 10^−2^	2.14 × 10^−2^	1.04 × 10^−2^
35	1.91 × 10^16^	1.34 × 10^−2^	1.39 × 10^−2^	3.60 × 10^−5^	4.83 × 10^−3^	2.81 × 10^−3^	7.93 × 10^−3^	1.18 × 10^−2^
36	1.06 × 10^17^	4.61 × 10^−10^	3.09 × 10^−10^	5.97 × 10^−6^	1.75 × 10^−5^	1.08 × 10^−2^	6.62 × 10^−3^	1.84 × 10^−4^
37	5.88 × 10^17^	1.27 × 10^−7^	7.26 × 10^−4^	1.44 × 10^−8^	7.90 × 10^−7^	3.15 × 10^−21^	2.79 × 10^−3^	3.15 × 10^−7^
38	3.26 × 10^18^	4.15 × 10^−8^	1.28 × 10^−2^	3.33 × 10^−6^	2.23 × 10^−4^	1.11 × 10^−2^	2.17 × 10^−3^	7.70 × 10^−11^
39	1.80 × 10^19^	6.61 × 10^−7^	6.24 × 10^−16^	6.18 × 10^−9^	1.67 × 10^−4^	1.13 × 10^−3^	3.95 × 10^−4^	2.22 × 10^−3^
40	1.00 × 10^20^	8.50 × 10^−12^	1.48 × 10^−2^	1.82 × 10^−4^	2.40 × 10^−8^	1.41 × 10^−2^	1.04 × 10^−3^	2.93 × 10^−5^

**Table 5:** The parameters of the finite element mesh.

Number of Elements by Sliding Layer Thickness (N)	4	8	12	16	24	32	40
Minimum overall dimension of the element, mm	1.000	0.500	0.330	0.250	0.167	0.125	0.100

## Data Availability

The original contributions presented in this study are included in the article. Further inquiries can be directed to the corresponding author.

## Abbreviations

PTFE	Polytetrafluoroethylene
UHMWPE	Ultra-high-molecular-weight polyethylenes
CAE	Computer-Aided Engineering
CTE	Coefficient of thermal expansion
DMA	Dynamic mechanical analysis
WLF	Williams–Landel–Ferry temperature–time analogy
MAPE	Mean absolute percentage error
RMSE	Root of the mean square error

## Appendix A

**Table A1:** The dependence of maximum stress intensity on temperature.

Material	Mathematical Relationship $\max\sigma_{\mathrm{int}}\left(T\right)$	R^2^	RMSE
PTFE	$\max\sigma_{\mathrm{int}}\left(T\right)=0.00288097\cdot T^{2}-0.3301014\cdot T+123.86012987$	0.9997	0.0396
F4Br40M2	$\max\sigma_{\mathrm{int}}\left(T\right)=0.00360025\cdot T^{2}-0.3843012\cdot T+126.1624975$	0.9988	0.0952
UHMWPE	$\max\sigma_{\mathrm{int}}\left(T\right)=0.00196553\cdot T^{2}-0.26333566\cdot T+120.85105894$	0.9996	0.0384
AR-200	$\max\sigma_{\mathrm{int}}\left(T\right)=0.00285549\cdot T^{2}-0.32943407\cdot T+123.80186813$	0.9998	0.0353
AR-202	$\max\sigma_{\mathrm{int}}\left(T\right)=0.00861119\cdot T^{2}-0.68456843\cdot T+137.32584416$	0.9967	0.2645
AR-204	$\max\sigma_{\mathrm{int}}\left(T\right)=0.00473118\cdot T^{2}-0.3170479\cdot T+101.51105794$	0.9059	0.6770
SF-1	$\max\sigma_{\mathrm{int}}\left(T\right)=0.00518052\cdot T^{2}-0.4917043\cdot T+130.38761239$	0.9983	0.1439

**Table A2:** The dependence of the maximum strain intensity on temperature.

Material	Mathematical Relationship $\max\varepsilon_{\mathrm{int}}\left(T\right)$	R^2^	RMSE
PTFE	$\max\varepsilon_{\mathrm{int}}\left(T\right)=0.00037793\cdot T^{2}-0.01466551\cdot T+7.01811489$	0.9968	0.0074
F4Br40M2	$\max\varepsilon_{\mathrm{int}}\left(T\right)=0.00039321\cdot T^{2}-0.01519178\cdot T+6.23571818$	0.9953	0.0093
UHMWPE	$\max\varepsilon_{\mathrm{int}}\left(T\right)=0.00034512\cdot T^{2}-0.01397718\cdot T+8.54650889$	0.9981	0.0052
AR-200	$\max\varepsilon_{\mathrm{int}}\left(T\right)=0.00037757\cdot T^{2}-0.01474905\cdot T+7.06867373$	0.9968	0.0074
AR-202	$\max\varepsilon_{\mathrm{int}}\left(T\right)=0.00039929\cdot T^{2}-0.01634739\cdot T+3.98644306$	0.9864	0.0161
AR-204	$\max\varepsilon_{\mathrm{int}}\left(T\right)=0.00037477\cdot T^{2}-0.00336578\cdot T+7.38279441$	0.9646	0.0339
SF-1	$\max\varepsilon_{\mathrm{int}}\left(T\right)=0.00040479\cdot T^{2}-0.01575569\cdot T+5.16483127$	0.9920	0.0125

**Table A3:** The dependence of the maximum of contact pressure $S_{{\mathrm{cont}}_{1}}$ on temperature.

Material	Mathematical Relationship $\max\;P_{\mathrm{cont}}\left(T\right)$	R^2^	RMSE
PTFE	$\max\;P_{\mathrm{cont}}\left(T\right)=0.00196824\cdot T^{2}-0.07984176\cdot T+77.04723077$	0.9714	0.1162
F4Br40M2	$\max\;P_{\mathrm{cont}}\left(T\right)=0.00240437\cdot T^{2}-0.07256109\cdot T+76.71681618$	0.9802	0.1228
UHMWPE	$\max\;P_{\mathrm{cont}}\left(T\right)=0.00120037\cdot T^{2}-0.0803038\cdot T+77.87541059$	0.9696	0.0941
AR-200	$\max\;P_{\mathrm{cont}}\left(T\right)=0.00194881\cdot T^{2}-0.08029565\cdot T+77.06744655$	0.9699	0.1183
AR-202	$\max\;P_{\mathrm{cont}}\left(T\right)=0.00545056\cdot T^{2}-0.19647183\cdot T+80.8741988$	0.8732	0.7199
AR-204	$\max\;P_{\mathrm{cont}}\left(T\right)=0.00203162\cdot T^{2}-0.14672483\cdot T+80.82003596$	0.9328	0.2617
SF-1	$\max\;P_{\mathrm{cont}}\left(T\right)=0.00370173\cdot T^{2}-0.10784166\cdot T+77.12033766$	0.9523	0.3001

**Table A4:** The dependence of the maximum of contact shear stress $S_{{\mathrm{cont}}_{1}}$ on temperature.

Material	Mathematical Relationship $\max\tau_{\mathrm{cont}}\left(T\right)$	R^2^	RMSE
PTFE	$\max\tau_{\mathrm{cont}}\left(T\right)=0.00008974\cdot T^{2}-0.00264417\cdot T+2.98857443$	0.9747	0.0052
F4Br40M2	$\max\tau_{\mathrm{cont}}\left(T\right)=0.0001093\cdot T^{2}-0.00269705\cdot T+2.99675005$	0.9818	0.0056
UHMWPE	$\max\tau_{\mathrm{cont}}\left(T\right)=0.00006713\cdot T^{2}-0.00312863\cdot T+2.99204505$	0.9313	0.0064
AR-200	$\max\tau_{\mathrm{cont}}\left(T\right)=0.0000888\cdot T^{2}-0.00265594\cdot T+2.9893029$	0.9728	0.0053
AR-202	$\max\tau_{\mathrm{cont}}\left(T\right)=0.00021802\cdot T^{2}-0.0078588\cdot T+3.23494905$	0.8732	0.0288
AR-204	$\max\tau_{\mathrm{cont}}\left(T\right)=0.00011825\cdot T^{2}-0.00605136\cdot T+3.03081658$	0.9160	0.0130
SF-1	$\max\tau_{\mathrm{cont}}\left(T\right)=0.00015797\cdot T^{2}-0.00449036\cdot T+3.05410599$	0.9259	0.0163

**Table A5:** The dependence of maximum normal displacement $S_{{\mathrm{cont}}_{3}}$ on temperature.

Material	Mathematical Relationship $\max u_{n}\left(T\right)$	R^2^	RMSE
PTFE	$\max u_{n}\left(T\right)=0.00001389\cdot T^{2}-0.00014475\cdot T+0.07742157$	0.9896	0.0007
F4Br40M2	$\max u_{n}\left(T\right)=0.00001459\cdot T^{2}-0.00015065\cdot T+0.06975374$	0.9866	0.0008
UHMWPE	$\max u_{n}\left(T\right)=0.00001263\cdot T^{2}-0.00013774\cdot T+0.09258692$	0.9932	0.0005
AR-200	$\max u_{n}\left(T\right)=0.00001385\cdot T^{2}-0.00014535\cdot T+0.07788543$	0.9897	0.0006
AR-202	$\max u_{n}\left(T\right)=0.00001683\cdot T^{2}-0.00017329\cdot T+0.04868684$	0.9734	0.0013
AR-204	$\max u_{n}\left(T\right)=0.00001167\cdot T^{2}-0.00020402\cdot T+0.09128698$	0.9859	0.0010
SF-1	$\max u_{n}\left(T\right)=0.00001557\cdot T^{2}-0.00015918\cdot T+0.05954626$	0.9808	0.0010
